# Supplementary material for: Extended Infusion of Meropenem in Neonatal Sepsis: A Historical Cohort Study
Source: Antibiotics (Basel). 2022 Mar 4;11(3):341. doi: 10.3390/antibiotics11030341 (PMC8944670; doi:10.3390/antibiotics11030341)
Supplement: Supplementary file 1 [file antibiotics-11-00341-s001.zip › antibiotics-1592241-supplementary.pdf]

antibiotics

MDPI

Article

Extended Infusion of Meropenem in Neonatal Sepsis: A Historical Cohort Study

Guangna Cao [1,†], Pengxiang Zhou [2,3,†], Hua Zhang [4], Bangkai Sun [5], Xiaomei Tong [1,*] and Yan Xing [1,*]

[1] Department of Pediatrics, Peking University Third Hospital, Beijing 100191, China; guangnacao@bjmu.edu.cn
[2] Department of Pharmacy, Peking University Third Hospital, Beijing 100191, China; pxzhou0427@bjmu.edu.cn
[3] Peking University Health Science Center, Institute for Drug Evaluation, Beijing 100191, China
[4] Research Center of Clinical Epidemiology, Peking University Third Hospital, Beijing 100191, China; zhanghua@bjmu.edu.cn
[5] Information Management and Big Data Center, Peking University Third Hospital, Beijing 100191, China; bangkaisun@bjmu.edu.cn
† These authors contributed equally to this article.
* Correspondence: tongxiaomei@bjmu.edu.cn (X.T.); xingyan@bjmu.edu.cn (Y.X.); Tel.: +86-010-8226-7671 (X.T.); +86-010-8226-7677 (Y.X.)

Supplementary Materials

Table S1 Baseline characteristics of VLBW neonates

Characteristic	EI group (n=86)	STI group (n=99)	P-values
Age (weeks), Mean (SD)	29.53±2.33	29.49±2.08	0.919
Weight (grams), Mean (SD)	1064.53±214.24	1071.31±235.29	0.839
Poor response	75 (87.2%)	79 (79.8%)	0.178
Antibiotics Upgrade	31 (36.05%)	40 (40.4%)	0.543
CRT extension	7 (8.1%)	15 (15.2%)	0.142
Hypothermia	7 (8.1%)	7 (7.1%)	0.784
Apnea	50 (58.1%)	54 (54.6%)	0.623
Dyspnea	49 (57.0%)	58 (58.6%)	0.825
Abdominal distention	25 (29.1%)	27 (27.3%)	0.786
Vomiting	2 (2.3%)	4 (4.0%)	0.810
Heart rate increase	36 (41.9%)	38 (38.4%)	0.630
Hypotension	5 (5.8%)	9 (9.1%)	0.401
Cool extremities	7 (8.1%)	14 (14.1%)	0.199
Jaundice	6 (7.0%)	11 (11.1%)	0.332
Scleroderma	4 (4.7%)	5 (5.1%)	1.000
Pale or Grey complexion	45 (52.3%)	42 (42.4%)	0.178
Convulsions	0 (0%)	4 (4.0%)	0.168

Note: EI, extended infusion; STI, short-term infusion; SD, standard deviation; CRT, capillary refill time.

Table S2 univariate analyses results of VLBW infants

Outcomes	EI group(n=86)	STI group (n=99)	P-values
3-day clinical efficacy rate	65 (75.6%)	56 (56.6%)	0.007
3-day microbial clearance	81 (94.2%)	84 (84.8%)	0.041
3-day CRP recovery rate	18 (20.9%)	14 (14.1%)	0.223
3-day WBC recovery rate	38 (44.2%)	42 (42.4%)	0.809

Note: VLBW, Very Low Birth Weight Infants; EI, extended infusion; STI, short-term infusion; CRP, C-reactive protein; WBC, white blood cell.

Table S3 multivariate analyses results of VLBW infants

Predictive factors	Odds Ratio (95% CI)	P-values	Odds Ratio (95% CI)	P-values
**Outcomes**	**3-day clinical efficacy rate**		**3-day microbial clearance**	
Infusion time	**0.39 (0.2, 0.78)**	**0.008**	5.51 (0.99, 30.65)	0.051
Age (weeks)	0.98 (0.82, 1.16)	0.796	**0.58 (0.37, 0.89)**	**0.013**
Weight (grams)	1 (1, 1)	0.505	1 (1, 1)	0.776
Poor response	0.04 (0, 1.35)	0.074	0.53 (0.09, 3.31)	0.500
Antibiotics Upgrade	0.57 (0.27, 1.18)	0.128	0.51 (0.1, 2.53)	0.406
CRT extension	0.87 (0.19, 3.99)	0.862	2.75 (0.17, 43.71)	0.474
Hypothermia	1.8 (0.42, 7.66)	0.426	4.29 (0.31, 60.13)	0.279
Apnea	0.7 (0.34, 1.44)	0.331	0.83 (0.16, 4.38)	0.830
Dyspnea	0.7 (0.35, 1.42)	0.323	0.44 (0.11, 1.81)	0.255
Abdominal distention	2.11 (0.91, 4.89)	0.081	1.93 (0.32, 11.7)	0.477
Vomiting	0.26 (0.04, 1.75)	0.166	16.76 (0.82, 344.21)	0.067
Heart rate increase	0.7 (0.34, 1.43)	0.326	1.03 (0.23, 4.65)	0.968
Hypotension	4.25 (0.69, 26.24)	0.119	**13.98 (1.31, 149.25**)	**0.029**
Cool extremities	0.53 (0.13, 2.15)	0.376	1.34 (0.11, 17.08)	0.820
Jaundice	1.19 (0.37, 3.86)	0.773	4.46 (0.4, 49.13)	0.222
Scleroderma	0.36 (0.08, 1.54)	0.169	**211.65** (**15, 2986.66**)	**< 0.001**
Pale or Grey complexion	1.16 (0.55, 2.42)	0.699	1.37 (0.29, 6.59)	0.694
Convulsions	0.48 (0.05, 5.13)	0.545	1.42 (0.04, 50.66)	0.849

Note: VLBW, Very Low Birth Weight Infants; CRT, capillary refill time. The bold figures indicate statistical differences

Table S4 Baseline characteristics of 2-h or 3-h EI group

Characteristic	2h EI group (n=67)	3h EI group (n=60)	p-values
Age (weeks), Mean (SD)	30.86 (29.07, 32.64)	30.71 (28.43, 33)	0.927
Weight (grams), Mean (SD)	1230 (1050, 1610)	1200 (940, 1705)	0.338
Poor response	60 (89.6%)	50 (83.3%)	0.304
Antibiotics Upgrade	18 (26.9%)	21 (35%)	0.321
CRT extension	5 (7.5%)	6 (10%)	0.612

**Characteristic**	**2h EI group (n=67)**	**3h EI group (n=60)**	**p-values**
Hypothermia	12 (18.0%)	11 (18.3%)	0.951
Apnea	31 (46.3%)	29 (48.3%)	0.816
Dyspnea	34 (50.8%)	33 (55%)	0.632
Abdominal distention	16 (23.9%)	23 (38.3%)	0.078
Vomiting	1 (1.5%)	3 (5%)	0.535
Heart rate increase	24 (35.8%)	21 (35%)	0.923
Hypotension	5 (7.5%)	2 (3.3%)	0.530
Cool extremities	4 (6.0%)	8 (13.3%)	0.157
Jaundice	12 (17.9%)	7 (11.7%)	0.325
Scleroderma	3 (4.5%)	3 (5%)	1.000
Pale or Grey complexion	27 (40.3%)	33 (55%)	0.098
Convulsions	0 (0.0%)	0 (0.0%)	NA

Note: EI, extended infusion; CRT, capillary refill time.

Table S5 univariate analyses results of 2-h or 3-h EI group

**Outcomes**	**2h EI group (n=67)**	**3h EI group (n=60)**	**P-values**
3-day clinical efficacy rate	57 (85.1%)	47 (78.3%)	0.325
3-day microbial clearance	63 (94.0%)	57 (95.0%)	>0.999
3-day CRP recovery rate	34 (50.7%)	33 (55.0%)	0.632
3-day WBC recovery rate	14 (20.9%)	16 (26.7%)	0.445

Note: EI, extended infusion; CRP, C-reactive protein; WBC, white blood cell.

Table S6 Judgment criteria of clinical effectiveness

Judgment criteria	Normal temperature (36.5℃ to 37.5 ℃)	Normal blood pressure	Normal heart rate	Non vasoactive drug use	Invasive mechanical ventilation is not required	No pale complexion or blooming	Good response	Normal CRT	No scleredema	Good intestinal tolerance	No jaundice	No vomiting	No abdominal distension
Clinical efficacy	√	√	√	√	√	√	√	√	√	±	±	±	±
Clinical non-efficacy	x	x	x	x	x	x	x	x	x	±	±	±	±

Note: The clinical effectiveness of each patient was judged by two newborn pediatricians (G.C. and Y.X.). CRT, capillary refill time
